# Topics of Public Interest

**Published:** 1911-11

**Authors:** 


### TOPICS OF PUBLIC INTEREST

### Proposed List of Important Medicaments

THE Council on Pharmacy and Chemistry of the A. M. A. has issued a circular letter to teachers of materia medica and therapeutics and members of examining boards, with a view to establishing a list of medicaments of real importance, to which examinations for license shall be confined. The ultimate object is to economize mental effort. A great deal of the anxiety of students is, rightly or wrongly, due to an impression that they must anticipate the hobbies of a particular examiner and that certain examiners will go out of their way to find an ancient herb, little used at present, with which to catch the unwary aspirant for a license.

We confess to a certain degree of sympathy with the student. We are quite certain, for example, that a favorite formula of Dr. Goodell's of which he naively said, hundreds of pills were used by his patients every spring, would have been denounced as an incompatibility by Dr. Wood. And we recollect a bit of faculty gossip to the effect that a famous surgeon told of the extreme ignorance of a student in regarding a cataract as fatty degeneration; whereupon the pathologist exclaimed "Why, John, that's what I taught him."

At any rate, there can be no dispute but that if the materia medica were printed in three books and if some Sibyl could burn two of them she would be justified in charging a far greater price for the remaining one than for the original three—always provided that the proper selection had been made. Let it be distinctly understood, however that we are thinking not primarily of the student's peace of mind and not at all of sparing him labor on materia medica and therapeutics. What interests us is the true economy of labor in developing a thorough knowledge of the use of drugs by concentrating on those essential and really valuable.

There is a fallacy, quite wide-spread, that one can practice medicine with ten or twenty drugs. To any one who holds this notion, we merely say, make a list of the armamentarium on hand in the office: alcohol, water, hydrogen peroxid, ether, chloroform, formaldehyd, ammonia, etc., etc., amounting to perhaps 50; count the contents of the hypodermic and emergency medical case, look over a year's file of favorite prescriptions. Even for a specialist, unless his work is extremely limited and mechanical, the list of drugs will easily reach a hundred. Still, unless a physician is continually reaching out for novelties and striving to be "original," he will probably not exceed 500 different drugs and, while the list will naturally change to correspond to improvements in the available supply, it may be considered that this number represents a full requirement for a license to practice.

It would seem a very simple matter to secure, from teachers and practitioners, a satisfactory list of useful and necessary drugs. But there is a surprising lack of unanimity of opinion when one passes the first hundred or so of drugs in almost universal use. For example,—and it should be understood that we resort to personal statements solely as an example—the writer would be willing to dispense with the entire list of opium preparations except one salt of morphine and of codeine, with physostigma and its derivatives and with many other standard favorites, yet he would plead for catnip, apocynum and dioscorea. A friend who has Long taught therapeutics and has been interested in the work of state boards and colleges, informs us that precisely such foolish notions are common among the very men who might be supposed to be in a position to choose most wisely from the vast array of the materia medica.

Neither does it seem that the choice can be limited to the Pharmacopoeia. However loyal we may be to official standards and however faithfully we may essay to follow the trite mixtures and shotgun formulae of the National Formulary—not overlooking its many sensible prescriptions—the great majority of us will, at the bed side, find occasion to prescribe many new chemic compounds, occasionally a discarded "time-honored" legacy from a few centuries ago, an ethical proprietary, and, once in a while, a preparation that we deride when on our feet at a medical meeting.

All things considered, it seems that the best compromise is to let each teacher use his own judgment but to limit formal examinations, especially for license, to an unquestioned list of indispensable drugs, listed in the Pharmacopoeia.

---

The Hygienic Crusade of the International Typographical Union.—Persons not directly concerned with labor in the narrow sense that it has come to have in the last few years, are inclined to think that a Union exists only to secure higher wages —which in the aggregate mean dollars of lower value—and fewer hours of labor. While this criticism may be somewhat justified in certain cases, the fact should not be overlooked that the chief objects of a labor organization should be to secure increased efficiency of labor and a betterment of social and hygienic conditions which react to the benefit of the community at large.

The International Typographical Union has advised and enforced many reforms along hygienic lines, destined not only to benefit its members by reducing the risk from tuberculosis, lead poisoning, and occupational disease generally, but employers by increasing the efficiency of labor and the whole country by the force of a good example. One of the most noteworthy of the achievements of this Union is the establishment of a Home at Colorado Springs, dating from 1892, originally intended for the aged and infirm, more recently developed by the addition of appropriate buildings, into a tuberculosis sanitarium.

By a substantial pension system, "It is the boast of the International Typographical Union that its members do not become public charges." The mental training and technical skill of printers brings them well within the average of many so-called professions and it is not surprising that they have manifested so broad and farsighted an intelligence. Visitors will be welcomed at the Home and guides will be furnished. We hope that any of our readers anticipating a trip to Colorado, will avail themselves of this invitation.

---

### The Commission Business

THE *New Zealand Medical Journal,* Aug. 1911, resumes a discussion started in November, as to the propriety of physicians' occupying rooms in connection with drug stores (chemists' shops in the original.) While the editor sagely remarks that such contiguity does not necessarily mean that the druggist acts as a tout for the physician and that this unethical practice may exist between a druggist and a physician not on the same premises, he plainly implies that it is well to avoid even the appearance of evil.

It seems, however, that New Zealand has already dealt radically with the secret commission evil, having "An Act for the Prohibition of Secret Commissions."

> "Clause 8.—Every person is guilty of an offence who advises any person to enter into a contract with a third person, and receives or agrees to receive from that third person, without the knowledge and consent of the person so advised, any gift or consideration as an inducement or reward for the giving of that advice, unless the person giving that advice was to the knowledge of the person so advised the agent of that third person."

Here is a practical, legal remedy, not only for medical fee splitting, but for touting of all kinds, in any line of business. Can we do better than copy this law in our own country?

